# Mechanism and empirical evidence on new-type urbanization to narrow the urban–rural income gap: Evidence from China’s provincial data

**DOI:** 10.1371/journal.pone.0270964

**Published:** 2024-08-02

**Authors:** Na Zhao

**Affiliations:** School of Marxism, Shaanxi Normal University, Xi’an, PR China; University of Almeria: Universidad de Almeria, SPAIN

## Abstract

The main component of China’s income gap is the urban–rural income gap, which is largely affected by urbanization. It is worth studying how new-type urbanization affects the income gap between urban and rural areas. Research mostly focuses on the urbanization rate as the core explanatory variable to explain the impact using one or two factors. This paper analyzes the mechanism of the effect using a comprehensive number of factors, with the quality of new-type urbanization development as the core explanatory variable. In terms of theoretical research, we believe that new-type urbanization affects the urban–rural income gap by promoting the transfer of labor, changing industrial structure, and policy tendency. Using both static and dynamic empirical analyses, we test the impact of new-type urbanization on the urban–rural income gap based on China’s provincial data. We find that new-type urbanization is conducive to narrowing the income gap between urban and rural areas. The transfer of labor significantly reduces the urban–rural income gap. However, the upgrading of industrial structure will enlarge the gap. The impact of China’s policy orientation is negligible. Policy should focus on promoting urbanization and improving the marginal rate of return of agriculture, improve the level of human capital, reverse the mismatch between employment structure and industrial structure, increase support for rural areas, and make substantial progress in promoting common prosperity.

## Introduction

The income gap between urban and rural areas in developing countries is largely affected by urbanization processes. A moderate income gap is conducive to an improvement in economic efficiency. However, an excessive gap is not only the embodiment of unfairness but also affects efficiency [1]. For example, in China—the world’s largest developing country—the main component of the income gap is the urban–rural income gap, which is largely affected by urbanization [2]. Traditional urbanization allow the urban–rural income gap to continue expanding in China. How the new-type urbanization affects the urban–rural income gap is the core content of this paper. There are three different views on the impact of urbanization on the urban–rural income gap. First, some researchers believe urbanization has widened the income gap between urban and rural areas. The research of García and Turnovsky has shown that differences in access to information, the biased allocation of resources, and fiscal policies have led to an expansion of the income gap between urban and rural areas [3]. Fujita et al. have found that the aggregation effect caused by the flow of population and production factors leads to faster income growth in urban areas than in rural areas, thus widening the income gap between urban and rural areas [4]. Second, the impact of urbanization on the urban–rural income gap presents an inverted U-shaped trajectory of first expanding and then narrowing. Robinson et al. have proved the inverted U-shaped hypothesis theoretically by using a two-sector model. Their research shows that when the transfer of rural labor reaches the Lewis turning point, the remuneration of agricultural labor begins to rise, and the income gap between urban and rural residents is bound to present an inverted U shape [5, 6]. Third, some studies have shown that urbanization has narrowed the gap between urban and rural areas. Mehta and Hasan demonstrated that the service industry can absorb a large portion of rural labor, which is conducive to improving the income of rural residents and narrowing the income gap between urban and rural areas [7]. Song et al. used the new economic geography (NEG) framework to analyze the impact of a heterogeneous rural labor transfer. Their results showed that labor transfer narrowed the income gap between urban and rural areas [8].

Most Chinese researchers have focused on the urbanization rate as the core explanatory variable and explain the impact using one or two factors. Some scholars believe that the policy of urban–rural segmentation comes from an urban bias in decision-making, the strategy of prioritizing the development of capital-intensive industries is one of the main reasons for the widening of China’s urban–rural income gap [9], it is believed that new-type urbanization will narrow the income gap between urban and rural areas [10]. Cheng’s research has shown that in the process of urbanization, the impact of rationalization and the upgrading of industrial structure on the urban–rural income gap is significantly different [11]. Some empirical studies have shown that government-led development has significantly widened the income gap between urban and rural areas, whereas population flow has narrowed it [12]. The proportion of the agricultural population, and the ratio of agricultural and non-agricultural labor productivity in urbanization are the long-term determinants of the urban–rural income gap [13].

Existing research on the impact of urbanization on urban-rural income gap shows diversified conclusions of expanding, narrowing, expanding first and then narrowing. It also shows the fragment mechanism analysis based on a single factor, and the simple value urbanization rate is mainly selected as the core explanatory variable. In 2014, China issued the national new-type urbanization plan (2014–2020) which proposed to comprehensively improve the quality of urbanization development. New-type urbanization is essentially different from traditional quantitative urbanization, it is an urbanization process of people-oriented, urban-rural planning, intensive-efficient, and advocating fair sharing. Since the release of the plan, China has continuously liberalized the restrictions on urban registered residence in the implementation of the new-type urbanization policy which has promoted the urban transfer of agricultural population and increased the supply of urban labor. Based on the particularity of China’s system and national conditions, this paper attempts to clarify the mechanism of the impact of new-type urbanization on urban–rural income gap. On the one hand, we synthesizes the impact mechanism of fragmentation, puts forward the research hypothesis that new-type urbanization affects the urban-rural income gap through labor transfer, industrial structure and policy tendency, and uses dynamic and static estimations to test the impact of new-type urbanization on the urban–rural income gap in China. On the other hand, different from previous studies with urbanization rate as the core explanatory variable, this study adopts the comprehensive measurement results of new-type urbanization as the core explanatory variable. In terms of structural arrangement, we first conduct mechanism analysis, then carry out empirical estimation including benchmark analysis, regional heterogeneity analysis and robustness test, and finally discuss and summarize the research conclusions.

## Mechanism analysis

We hypothesize that new-type urbanization affects the urban–rural income gap by promoting labor transfer, upgrading industrial structure, and changing policy orientation based on classical theory and literature analysis.

### Narrowing the gap by accelerating the transfer of the labor force

Hypothesis 1: **Society is composed of urban and rural sectors**, labor enjoys a high degree of mobility, and the natural growth rate of the population is zero. *Lu* and *Lr* represent urban population and rural population respectively, *Iu* and *Ir* represent urban per capita income and rural per capita income respectively. Then the total number of population *L* is *L = Lu+Lr*.

Hypothesis 2: Considering the change in wages and population size caused by dynamic population transfer, we set the urban and rural income growth rates as *θ*_*u*_ and *θ*_r_, where *θ*_u_>*θ*_r_. Due to the registered residence system barrier of China’s urban-rural division in traditional urbanization, China’s rural labor force is not divided into urban and rural population as other countries, but into the following three types in the dynamic transfer of urban and rural areas: (1) urban population: the ratio of the population that has obtained registered urban residency and employment opportunities, denoted as *m*; (2) semi urban population: the ratio of the population that has urban employment opportunities but has not obtained registered urban residency, denoted as *n*, and their corresponding income growth rate is *θ*_*ur*_; and (3) farmers: the ratio of the labor force engaged in agricultural production, denoted as *a*. We construct a two-sector model to analyze the urban–rural income gap *I*_*ur*0_ in the initial stage based on the above assumptions [14]:

$$I_{ur0}=I_{u0}/I_{r0}$$ (1)

With the continuous and dynamic transfer of rural surplus labor force, the size of the urban population and per capita income change is

$$I_{u1}=I_{u0}(1+\theta_{u})$$ (2)

Changes in rural income are

$$I_{r1}=I_{r0}\cdot \frac{(1+\theta_{r})a+(1+\theta_{ur})n}{a+n}$$ (3)

Changes in the urban–rural income gap can be depicted as

$$I_{ur1}=\eta \cdot I_{ur0}=\frac{I_{u0}}{I_{r0}}\cdot \frac{\left(1+\theta_{u})(a+n\right)}{(1+\theta_{r})a+(1+\theta_{ur})n}$$ (4)

The coefficient of the change in the urban–rural income gap is

$$\eta =\frac{\left(1+\theta_{u})(a+n\right)}{(1+\theta_{r})a+(1+\theta_{ur})n}$$ (5)

If the change coefficient of urban-rural income gap η > 1, the relative urban–rural income gap increases; when η = 1, there is no change in the income gap; and when η < 1, the income gap narrows. The conditions for dynamic convergence of η are as follows. First, the increase in the size of the agricultural transfer population with non-urban registered residency increases the average income level of the rural area but is not integrated into the urban area. Second, *θ*_*r*_ and *θ*_*ur*_ increase if *θ*_*u*_>*θ*_*ur*_>*θ*_*r*_, and when the growth rate in urban income levels is higher than that of rural and semi-urban regions, the income gap between urban and rural areas will continue to expand. When *θ*_*u*_ is constant, *θ*_*r*_ and *θ*_*ur*_ increase, and the urban–rural income gap tends to converge. With the development of new-type urbanization, China is continuously liberalizing the restrictions on registered residence, the semi urbanized population “n” who has worked and lived in cities for a long time without urban registered residence is decreasing until tends to 0, n = 0 in the long run. New-type urbanization increases the urban labor supply and decreases the urban income growth rate. Semi urbanization is bound to decrease, and the rural income growth rate will continue to rise. At the same time, agricultural productivity will improve and the agricultural income growth rate will also improve. Lastly, urban and rural factor rewards tend to be equal, and the urban–rural income gap narrows, forming a new coefficient of change:

$$\eta_{new}=\frac{1+\theta_{u}}{1+\theta_{r}}$$ (6)

### Influence of promoting the upgrading of industrial structure

**Fig 1:**
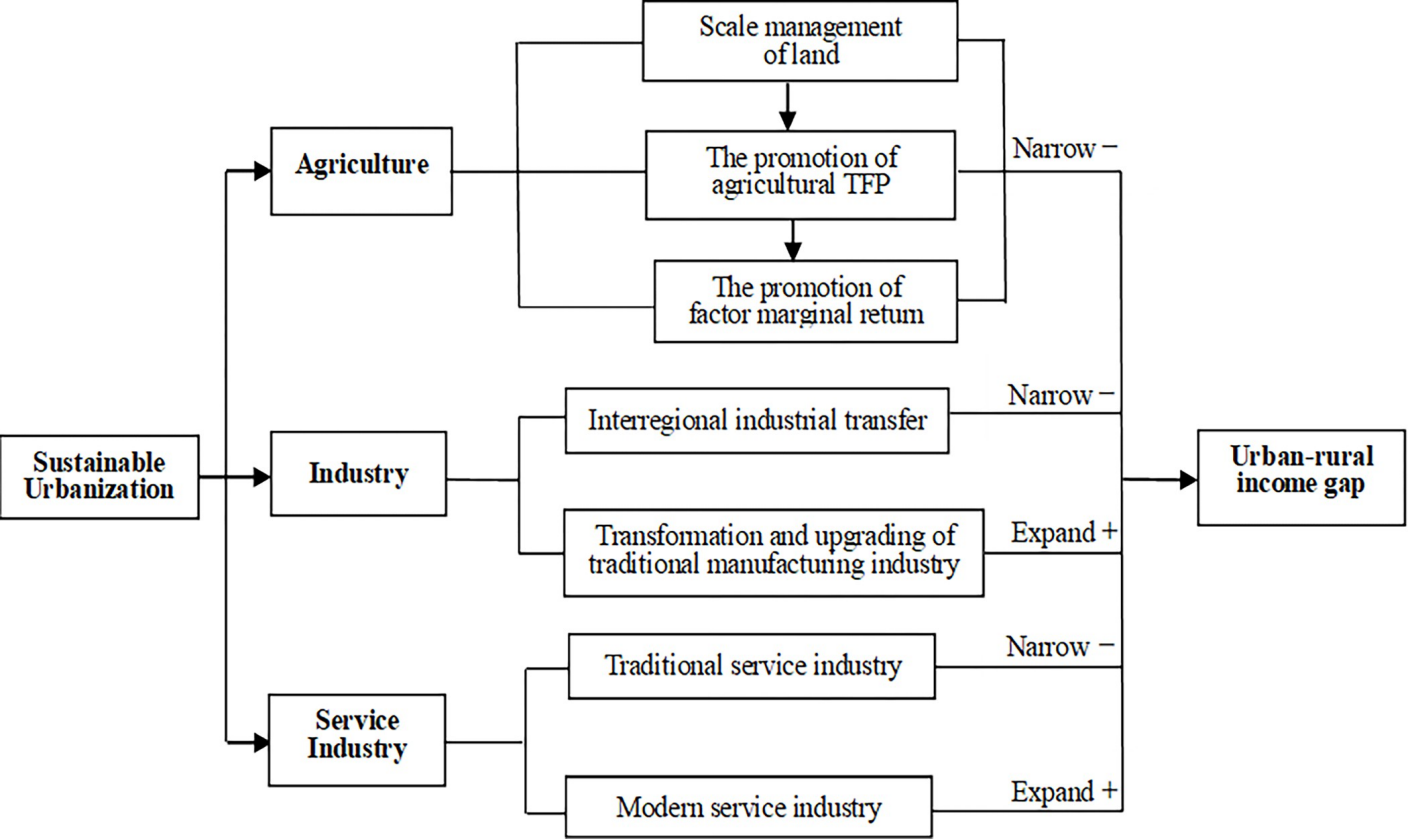
Influenced by promoting the upgrading of industrial structure.

Industrial structure will change in terms of quality and innovation in new-type urbanization, and develop an industrial pattern dominated by modern agriculture, high-end manufacturing, and modern service industries [15]. The change in industrial structure is consistent with urbanization, and the industrial structure will be further optimized and upgraded during new-type urbanization. First, with the implementation of China’s “separation of three powers” policy, large-scale land management promotes agricultural modernization in new-type urbanization. Second, industrial transfer can absorb agriculture’s surplus labor force both locally and in neighboring regions, which is conducive to narrowing the urban–rural income gap. The transformation and upgrading of traditional manufacturing industries to high-end manufacturing industries will reduce the demand for low-end labor, which may expand the urban–rural income gap. Third, the employment scale of the service industry is large and stable, which is conducive to narrowing the urban–rural income gap. The modern service industry has a high rate return for labor, but its high technology and high human capital requirements serve as entry barrier to the low-end labor force (Fig 1).

### Narrowing the gap by changing policy orientation

Urban or rural policy bias also greatly affects the urban–rural income gap. For example, a strategy of prioritizing the development of urban areas will allocate a higher proportion of local fiscal expenditure to cities. A two-sector model is constructed to develop the welfare function as follows:

$$W=W(U,R)=W(U(G_{U}),\;R(G_{R}))$$ (7)

The output of the urban sector is *U*, the rural sector output is *R*, and the government public expenditure is *G*. *G*_*U*_ and *G*_*R*_ are urban public expenditure and rural public expenditure, respectively, where *G*_*U*_ > 0, and *G*_*R*_ > 0. The optimal social welfare function satisfies:

$$Max\;W=W(U(G_{U}),\;R(G_{R}))$$ (8)

$$s.t.\;G=G_{u}+G_{R}$$ (9)

The corresponding Lagrange function is:

$$L=W(U(G_{U}),R(G_{R}))-\lambda (G-G_{u}-G_{R})$$ (10)

The partial derivatives calculated for *G*_*U*_ and *G*_*R*_ are:

$$\frac{\partial W}{\partial U}\cdot \frac{\partial U}{\partial G_{U}}=\frac{\partial W}{\partial R}\cdot \frac{\partial R}{\partial G_{R}}$$ (11)

National welfare depends on the distribution ratio of government public expenditure in the two sectors. The elasticity coefficient of urban output welfare is $E_{W,U}=\frac{\partial W/W}{\partial U/U}$, the elasticity coefficient of rural output welfare is $E_{W,R}=\frac{\partial W/W}{\partial R/R}$, the output elasticity coefficient of urban public expenditure is $E_{U,G_{U}}=\frac{\partial U/U}{\partial G_{U}/G_{U}}$, and the corresponding rural coefficient is $E_{R,G_{R}}=\frac{\partial R/R}{\partial G_{R}/G_{R}}$. It can be concluded that:

$$\frac{G_{U}}{G_{R}}=\frac{E_{W,U}}{E_{W,R}}\cdot \frac{E_{U,G_{U}}}{E_{R,G_{R}}}$$ (12)

$$\frac{E_{W,U}}{E_{W,R}}=\lambda \frac{U}{R}$$ (13)

λ>0:

$$\frac{U}{R}=\frac{1}{\lambda }\cdot \frac{G_{U}}{G_{R}}\cdot \frac{E_{R,G_{R}}}{E_{U,G_{U}}}$$ (14)

The urban–rural income gap is proportional to the government’s public expenditure in the two sectors [16]. Increasing or reducing rural public expenditure is conducive to narrowing or expanding the income gap between urban and rural areas if the urban public expenditure is fixed, and the reverse is also true.

## Model construction and empirical test

### Data gathering and variable selection

China’s four major regions include the East, northeast, central and western regions, of which the eastern region includes 10 provinces of Beijing, Tianjin, Hebei, Shanghai, Jiangsu, Zhejiang, Fujian, Shandong, Guangdong and Hainan. Northeast China includes Liaoning, Jilin and Heilongjiang provinces. The central part includes six provinces: Shanxi, Anhui, Jiangxi, Henan, Hubei and Hunan. The West includes 12 provinces in Inner Mongolia, Guangxi, Chongqing, Sichuan, Guizhou, Yunnan, Tibet, Shaanxi, Gansu, Qinghai, Ningxia and Xinjiang. Due to the limitations of data acquisition, this study does not include Tibet. Panel data of 30 provinces were extracted from the China Statistical Yearbook, provincial statistical yearbooks, the China Economic and Social Development Statistics database, the China Population and Employment Statistical Yearbook, and the Wind database from 2009 to 2018. The meaning and descriptions of the model’s variables are shown in Table 1:

**Table 1:** Descriptions of the variables.

Variable Category	Symbol	Variable Name	Specific Description
Explained	GT	Urban-rural income gap	Measure with Theil index
Core Explanatory	UQ	New-type urbanization	Measurement score of new-type urbanization
Explanatory	MR	Labor transfer	The proportion of non-agricultural employment in total
	SR	Rational of industrial	Measure with Theil index
	SH	Upgrading of industrial	The ratio of service industry to industrial output
	PB	Policy orientation	Financial expenditure on agriculture, forestry, and water
Control	PGDP	Economic Growth	Per capita GDP
	EDU	Investment in education	The proportion of education in financial expenditure
	PK	Investment in fixed assets	Per capita investment in fixed assets

We use *y*_*ut*_ and *y*_*rt*_ to represent the total income of urban and rural residents in year *t*. The total income of residents is *y*_*t*_ in that year, and the size of the urban and rural population is *s*_*ut*_ and *s*_*rt*,_ respectively. The total population of urban and rural areas is *s*_*t*_ [17]. The province’s Theil index for the urban–rural income gap in year *t* is T_*urt*_:

$$T_{urt}=\frac{y_{ut}}{y_{t}}\ln\left[\left(\frac{y_{ut}}{y_{t}}\right)/\frac{s_{ut}}{s_{t}}\right]+\frac{y_{rt}}{y_{t}}\ln\left[\left(\frac{y_{rt}}{y_{t}}\right)/\frac{s_{rt}}{s_{t}}\right]$$ (15)

The explanatory variable UQ represents the development quality of new-type urbanization, measurement for new-type urbanization includes seven economic indicators, six innovation indicators, seven coordination indicators, seven green indicators, five open indicators, and 10 sharing indicators. Overall, 42 indicators are measured using an improved entropy method, and detailed measurement results are presented both in the author’s published literature and appendices of this paper due to the length limitation [18]. There are 42 indicators selected for UQ measure, and its redundancy is very low. It can also be seen from the attached table that the weight of each indicator is very small, and the correlation with other variables can be ignored. The industrial structure rationalization of explanatory variables is measured by the Theil index. The specific sectors of the three industries are represented by *i*; *Y* is total output, *Yi* is the output of *i* industry, *L* is total employment, and *Li* is employment in *i* industry:

$$SR=\sum_{i=1}^{3}\frac{Y_{i}}{Y}ln(\frac{Y_{i}}{L_{i}}/\frac{Y}{L})$$ (16)

The upgrading of industrial structure is measured by the ratio of the output value of the tertiary industry to the secondary industry [19]. The natural logarithms of *UQ*, *Pb*, *PGDP*, and *PK* are used to estimate the model for eliminating the heteroscedasticity of the relevant data.

### Model construction

Taking other factors that may affect the urban–rural income gap as control variables, this paper constructs the following static panel model:

$${GT}_{it}=\alpha_{0}+\alpha_{1}{UQ}_{it}+\alpha_{2}{MR}_{it}+\alpha_{3}{SR}_{it}+\alpha_{4}{SH}_{it}+\alpha_{5}{PB}_{it}+\beta_{1}{PGDP}_{it}+\beta_{2}{EDU}_{it}+\beta_{3}{PK}_{it}+\xi_{it}$$ (17)

The section unit number is *i*, *t* represents the year, and *ξ* is the residual term. The income gap between urban and rural areas is also affected by the value of the previous period. It is, therefore, necessary to consider dynamic change in the empirical analysis. The dynamic panel model introduces a lag of one period in the urban–rural income gap as follows:

$${GT}_{it}=\alpha_{0}+{+\theta {GT}_{it-1}+\alpha }_{1}{UQ}_{it}+\alpha_{2}{MR}_{it}+\alpha_{3}{SR}_{it}+\alpha_{4}{SH}_{it}+\alpha_{5}{PB}_{it}+\beta_{1}{PGDP}_{it}+\beta_{2}{EDU}_{it}+\beta_{3}{PK}_{it}+u_{i}+\xi_{it}$$ (18)

*GT*_*it*−1_ is the lag term of *GT*_*it*_, *u*_*i*_ represents the intercept term of individual heterogeneity, and *u*_*i*_ and *ξ*_*it*_ constitute compound disturbance terms.

### Benchmark regression

Sort out the data, Table 2 shows the descriptive statistics of variables:

**Table 2:** Variable descriptive statistics.

Variable	NO. of samples	Mean value	Std. Deviation	Min	Max
GT	300	0.109	0.051	0.020	0.257
lnUQ	300	3.012	0.801	1.531	5.135
MR	300	0.640	0.149	0.289	0.969
SR	300	0.298	0.177	0.029	0.826
SH	300	1.020	0.603	0.381	4.237
lnPB	300	5.777	0.669	3.652	6.916
lnPGDP	300	10.575	0.512	9.196	11.768
EDU	300	0.180	0.028	0.111	0.282
lnPK	300	10.216	0.536	8.517	11.312

### Static regression analysis

A variety of methods are used for regression to ensure the robustness of the estimation results. First, a least square dummy variable (LSDV) estimation with individual dummy variables is used; models I and II are the estimation results without and with control variables. A feasible generalized least squares (FGLS) estimation is used as the disturbance term may have intragroup autocorrelation or intergroup heteroscedasticity. Lastly, according to the results of the Hausman Test, we use the fixed-effects model to estimate. As shown in Table 3, the three estimation methods all verify the convergence effect of China’s new-type urbanization on the urban–rural income gap, and the influence coefficient is relatively close. All other things being equal, the income gap will decrease by 0.0526 units for every unit of new-type urbanization improvement based on the estimation results of the fixed-effects model with control variables [20]. The static regression results show that the upgrading of industrial structure will expand the urban-rural income gap. It may be that the rationalization and upgrading of industrial structure tend to attract more labor forces with higher human capital levels, they are easy to obtain urban registered residence, and counted as urban labor forces in data statistics. The coefficient of policy orientation remains small, whether the control variable is added or not. It shows that China’s policy tendency for rural areas has not had a substantive impact on narrowing the gap between urban and rural areas. An increase in investment in fixed assets will narrow the income gap, it may be that fixed asset investment requires a large number of semi urbanized people, such as migrant workers for infrastructure construction, in which brings employment opportunities to the transferred population and improves their income. The influence of economic growth and investment in education is not significant. Table 3 shows the static regression results:

**Table 3:** Static regression results.

Variable	LSDV		FGLS		FE	
	Ⅰ	Ⅱ	Ⅲ	Ⅳ	Ⅴ	Ⅵ
lnUQ	-0.0738***	-0.0526***	-0.0700***	-0.0362***	-0.0738***	-0.0526***
	(0.0059)	(0.0077)	(0.0040)	(0.0074)	(0.0105)	(0.0131)
MR	-0.0052	0.0546	0.0068	0.0692*	-0.0052	0.0546
	(0.0485)	(0.0472)	(0.0203)	(0.0376)	(0.0792)	(0.0624)
SR	0.0403***	0.0391**	0.0455***	0.0663***	0.0403**	0.0391**
	(0.0155)	(0.0160)	(0.0083)	(0.0108)	(0.0169)	(0.0176)
SH	0.0257***	0.0157***	0.0166***	0.0091***	0.0257***	0.0157***
	(0.0039)	(0.0037)	(0.0020)	(0.0025)	(0.0066)	(0.0043)
lnPB	-0.0007	0.0096**	-0.0003	0.0057*	-0.0007	0.0096**
	(0.0036)	(0.0039)	(0.0028)	(0.0034)	(0.0045)	(0.0042)
lnPGDP		-0.0115		-0.0253***		-0.0115
		(0.0102)		(0.0071)		(0.0093)
EDU		-0.0697*		-0.0078		-0.0697
		(0.0403)		(0.0257)		(0.0493)
lnPK		-0.0194***		-0.0089***		-0.0194***
		(0.0064)		(0.0021)		(0.0082)
R-squared	0.970	0.974			0.845	0.866

Note: 1. The standard error in brackets; 2. * * *p<0.01

* * p<0.05

* p<0.1

### Dynamic regression analysis

One-step and two-step differential generalized method of moments (GMM) estimations are carried out by introducing a dynamic model with a delay term [21]. The *P* values of AR (1) and AR (2) are 0.000 and 0.224, respectively. The difference in perturbations has a first-order autocorrelation, but there is no second-order autocorrelation. A Sargan test shows that the *p*-value of over recognition is 0.799, and a differential GMM estimation can be used. Table 4 shows the dynamic regression results:

**Table 4:** Dynamic regression results.

Variable	GMM-1		GMM-2	
	Ⅰ	Ⅱ	Ⅲ	Ⅳ
L.GT	0.607***	0.553***	0.602***	0.549***
	(0.0696)	(0.0704)	(0.0090)	(0.0228)
lnUQ	-0.0262***	-0.0263**	-0.0229***	-0.0260***
	(0.0088)	(0.0116)	(0.0025)	(0.0065)
MR	-0.0758	-0.0749	-0.111***	-0.112**
	(0.0553)	(0.0576)	(0.0245)	(0.0480)
SR	0.0044	-0.0095	0.0015	-0.0109
	(0.0191)	(0.0190)	(0.0055)	(0.0159)
SH	0.0073*	0.0020	0.0063***	0.0023
	(0.0043)	(0.0046)	(0.0009)	(0.0014)
lnPB	0.0017	0.0047	0.0018*	0.0037**
	(0.0055)	(0.0056)	(0.0011)	(0.0017)
lnPGDP		-0.0017		0.0037
		(0.0133)		(0.0049)
EDU		-0.135***		-0.134***
		(0.0373)		(0.0181)
lnPK		-0.0049		-0.0053**
		(0.0040)		(0.0021)

Note: 1. The standard error in brackets; 2. * * *p<0.01

* * p<0.05

* p<0.1.

The results of the two-step differential GMM regression show that new-type urbanization converges the urban–rural income gap at a significant level of 1%. Models Ⅰ to Ⅳ show that the urban–rural income gap lagging one period has a significant impact on the current period. The previous period will expand the current urban–rural income gap, and the change in the urban–rural income gap has certain sustainability. The urban–rural income gap narrows by 0.026 units for every unit improvement in the quality of new-type urbanization with the addition of control variables.

## Regional heterogeneity

### Static regression analysis

Static regression results in Table 5 show that new-type urbanization in the eastern regions of China hurts the urban–rural income gap, but the effect is not significant. The upgrading of industrial structure in eastern China will widen the urban–rural income gap, it may be that the industrial structure of the eastern region is more advanced and requires a higher level of education for the transferred labor force. An increase in investment in fixed assets in eastern China is conducive to narrowing the regional income gap between urban and rural areas. The results for China’s central and northeastern regions are not significant. New-type urbanization in western China is conducive to narrowing the income gap between urban and rural areas. The income gap converges by 0.055% when the regional new-type urbanization increases by 1%, most western provinces are at a low level of urbanization, and the promotion of urbanization has significantly narrowed the urban-rural income gap.

**Table 5:** Static regression results by region: FEM.

Variable	China	East	Northeast	Central	West
lnUQ	-0.0526***	-0.0168	-0.0219	-0.0576	-0.0547***
	(0.0131)	(0.0162)	(0.0348)	(0.0287)	(0.0136)
MR	0.0546	-0.105	-0.0783	0.102	0.0334
	(0.0624)	(0.0802)	(0.0427)	(0.275)	(0.0805)
SR	0.0391**	0.134	-0.0386	-0.0030	0.0011
	(0.0176)	(0.107)	(0.0295)	(0.0400)	(0.0350)
SH	0.0157***	0.0135***	0.0030	-0.0114	0.0083
	(0.0043)	(0.0039)	(0.0040)	(0.0087)	(0.0098)
lnPB	0.0096**	0.0048	-0.0014	-0.0052	0.0134
	(0.0042)	(0.0041)	(0.0101)	(0.0119)	(0.0078)
lnPGDP	-0.0115	0.0094	-0.0242	0.0045	-0.0247
	(0.0093)	(0.0129)	(0.0365)	(0.0245)	(0.0222)
EDU	-0.0697	0.0001	-0.0967	-0.155	-0.219
	(0.0493)	(0.0278)	(0.118)	(0.0852)	(0.163)
lnPK	-0.0194**	-0.0280**	0.0050	-0.0058	-0.0217
	(0.0082)	(0.0091)	(0.0047)	(0.0055)	(0.0124)
R-squared	0.866	0.923	0.865	0.907	0.920

Note: 1. The standard error in brackets; 2. * * *p<0.01

* * p<0.05

* p<0.1.

### Dynamic regression analysis

A number of conclusions can be drawn from the dynamic regression analysis. First, Table 6 shows that new-type urbanization in China’s eastern regions has a positive effect on the urban–rural income gap, but the coefficient is small and not significant. The urban–rural income gap narrows by 0.379% for every 1% increase in labor transfer and increases by 0.01% for every 1% increase in industrial structure upgrading. Second, new-type urbanization in northeastern China has a negative effect. For every 1% increase in labor transfer, the regional urban–rural income gap converges by 0.203%. Third, new-type urbanization in the central regions of China converges the urban–rural income gap. This income gap converges by 0.077% for every 1% increase in new-type urbanization if other conditions remain unchanged. The gap increases by 0.074% when economic development increases by 1%, and converges by 0.171% when the investment in education increases by 1%. Lastly, if new-type urbanization in the western regions of China increases by 1%, the urban–rural income gap will converge by 0.038%.

**Table 6:** Results of regional dynamic regression: GMM.

Variable	China	East	Northeast	Central	West
L.GT	0.553***	0.211*	0.421**	0.670***	0.343***
	(0.0704)	(0.117)	(0.192)	(0.153)	(0.122)
lnUQ	-0.0263**	0.0090	-0.0375	-0.0768**	-0.0380*
	(0.0116)	(0.0118)	(0.0273)	(0.0346)	(0.0203)
MR	-0.0749	-0.379***	-0.203***	0.155	0.0173
	(0.0576)	(0.0954)	(0.0677)	(0.165)	(0.0859)
SR	-0.0095	-0.0417	-0.0413	-0.0036	0.0032
	(0.0190)	(0.0717)	(0.0312)	(0.0710)	(0.0288)
SH	0.0020	0.0103**	-0.0054	0.0121	-0.0120
	(0.0046)	(0.0048)	(0.0087)	(0.0170)	(0.0104)
lnPB	0.0047	-0.0059	0.0176	-0.0246	0.0175
	(0.0056)	(0.0045)	(0.0110)	(0.0186)	(0.0113)
lnPGDP	-0.0017	0.0001	0.0004	0.0742*	-0.0357
	(0.0133)	(0.0145)	(0.0159)	(0.0419)	(0.0245)
EDU	-0.135***	-0.0319	-0.149	-0.171*	-0.343***
	(0.0373)	(0.0243)	(0.114)	(0.100)	(0.0944)
lnPK	-0.0049	-0.0131	0.0049	-0.0023	-0.0053
	(0.0040)	(0.0092)	(0.0048)	(0.0074)	(0.0099)

Note: 1. The standard error in brackets; 2. * * *p<0.01

* * p<0.05

* p<0.1.

### Robustness test

We use the urban–rural income ratio to replace the Theil index of the urban–rural income gap to test the stability of the model and to ensure that the estimation results are not affected by the measurement methods. The ratio of urban–rural income *I*_*urt*_ is the ratio of per capita disposable income of urban *I*_*ut*_ to the per capita net income of rural *I*_*rt*_ [18, 22].The estimated results are shown in Table 7.


$$I_{urt}=I_{ut}/I_{rt}$$ (19)

**Table 7:** Robustness test: Urban–rural income ratio.

Variable	FEM		GMM	
	Ⅰ	Ⅱ	Ⅲ	Ⅳ
L.GT			0.445***	0.431***
			(0.0052)	(0.0266)
lnUQ	-0.811***	-0.593***	-0.277***	-0.270***
	(0.136)	(0.196)	(0.0308)	(0.0894)
MR	-0.0790	0.574	-1.499***	-1.422***
	(1.068)	(0.830)	(0.347)	(0.503)
SR	0.465**	0.447*	-0.0072	-0.174
	(0.204)	(0.234)	(0.116)	(0.146)
SH	0.326***	0.217***	0.138***	0.0511***
	(0.0924)	(0.0625)	(0.0152)	(0.0131)
lnPB	-0.0022	0.109*	-0.0562***	-0.0219
	(0.0509)	(0.0574)	(0.0101)	(0.0193)
lnPGDP		-0.0852		-0.0073
		(0.127)		(0.0711)
EDU		-0.752		-1.705***
		(0.644)		(0.201)
lnPK		-0.229**		-0.0314
		(0.0922)		(0.0247)
R-squared	0.779	0.800		

Note: 1. The standard error in brackets; 2. * * *p<0.01

* * p<0.05

* p<0.1.

## Discussion

Does new-type urbanization narrow the income gap between urban and rural areas? The results of our dynamic and static regression analyses and robustness test show that China’s new-type urbanization development significantly converged the urban–rural income gap from 2009 to 2018. In general, new-type urbanization development is conducive to convergence in the urban and rural income gap. The research contributions may be summarized as follows. (1) The income gap between urban and rural areas in the previous period will expand the said gap in the current period, and the change has a degree of sustainability. (2) Labor transfer will significantly narrow the income gap between urban and rural areas consistent with the mechanism. (3) Upgrading industrial structure will increase the income gap between urban and rural areas, possibly because the higher level of industrial structure will attract labor with higher human capital. This part of the population will settle down in large, registered residences in cities and towns, and the urban–rural income gap will widen statistically. (4) Policy orientation has a significant positive effect on the income gap between urban and rural areas, but the value of the coefficient is small, which may be because of the long-term nature of China’s urban policies and a degree of inertia. (5) Lastly, among the control variables, increasing investment in education and fixed assets are conducive to convergence in the urban–rural income gap [23–25]. Increasing investment in fixed assets will narrow the income gap between urban and rural areas, partly because the investment in fixed assets will mostly be in infrastructure construction that requires a large number of migrant workers, and these employment opportunities will improve their income level.

From a regional perspective, we can draw the following conclusions. (1) When the urban–rural income gap in the four regions lags by one period, it has a significant impact on the current urban–rural income gap; however, the degree of influence in each region reflects the heterogeneity law of weakening from the central to the northeastern regions, and from the west and to the east. (2) The impact of new-type urbanization on regional urban–rural income gaps is not significant in the eastern regions of China, which may be because of the higher level of urbanization and the fact that eastern regions are in a late stage of urbanization. In this stage, urbanization development is slow, and a trend of counter urbanization may appear; thus, the impact is relatively weak. New-type urbanization in China’s central and western regions will significantly narrow the income gap between urban and rural areas. (3) The transfer of the labor force in eastern and northeastern China significantly converges the income gap between urban and rural areas. (4) The upgrading of industrial structure has widened the income gap between urban and rural areas in the eastern regions, possibly because of their relatively developed economies, which have higher requirements for the level of human capital. (5) Among the control variables, the level of economic development in China’s central regions will increase the urban–rural income gap, and the increase in investment in education in the central and western regions will significantly converge the regional urban–rural income gap.

## Conclusions

This study combines classic theory and literature analysis, examines the specific mechanism. Different from previous studies on the fragmentation analysis of the mechanism of traditional urbanization on income gap, we believes that new-type urbanization affects the urban–rural income gap by promoting labor transfer “MR”, upgrading industrial structure “SR” & “SH”, and policy orientation “PB”, and taking them as the explanatory variables, new-type urbanization development as the core explanatory variable, We have made some new discoveries through mechanism analysis and dynamic and static empirical analysis.

This study’s contribution is to further promote new urbanization and enhance the marginal rate of return of agriculture. Improving the income of rural residents is key to narrowing the income gap between urban and rural areas [26, 27]. The change in the Theil index and the urban–rural income ratio of the urban–rural income gap shows that although the current urban–rural income gap is shrinking, it remains large in absolute terms [28]. High income and access to good public service and social security are still attractive to the surplus labor force in rural areas. On the one hand, China should improve the registered residence system and actively promote full citizenship in the agricultural transfer population, so that they can enjoy equal social security and basic public services. On the other hand, China should intensify the land reform system, implement the land policy of “separation of the three rights,” promote agricultural modernization, enhance the marginal returns of primary industry, and narrow the income gap between urban and rural areas [29, 30].

Both dynamic and static analysis show that the upgrading of industrial structure in which include rational of industrial “SR” and upgrading of industrial “SH” will expand the urban-rural income gap. As analyzed in this paper, the upgrading of industrial structure needs more transfer population with higher education. At the same time, the increase of education investment in the control variables“EDU” is conducive to narrowing the urban-rural income gap. Therefore, increasing education investment, improving human capital in the process of new urbanization is particularly important. It is necessary to improve human capital in the agricultural transfer population and reverse the mismatch between employment structure and industrial structure. China’s traditional manufacturing industry’s ability to absorb the agricultural transfer population is decreasing, and there is a shortage in high-end talent and professional technical service talent in the tertiary industry. Human capital in China’s agricultural transfer population is generally low, and it cannot match the requirements of an advanced industrial structure. Therefore, there is an urgent need to increase education resources in rural areas. The empirical results also show that an increase in investment in education will significantly reduce the income gap between urban and rural areas, especially in China’s central and western regions, where the impact coefficient of education investment is higher. The promotion of human capital in the agricultural transfer population will allow it to adapt to the new economic structure, realize the balanced development of urban and rural human capital level, help narrow the income gap between urban and rural areas, and promote social equity.

China’s policies tend to show an urban bias and a degree of inertia, policy support for rural areas should be increased. The empirical analysis shows that policy orientation “PB” has a significant positive effect on the urban-rural income gap, but the value of the coefficient is small. Although China is implementing support policies to boost rural development, such as rural revitalization and land policy of three rights system, support needs to be further strengthened. Urban bias has a great impact on traditional urbanization processes in China. The policy effect of a greater rural bias will not appear quickly in the short term [31]. On the one hand, the proportion of financial support for agriculture remains relatively low and needs to be increased, especially in terms of direct benefits to farmers [32]. On the other hand, agricultural infrastructure construction and agricultural technology support should be increased to develop a modern agriculture and fundamentally improve total agricultural factor productivity.

## Supporting information

S1 Appendix (DOCX)

S1 Data (XLSX)
