# Correction: Mechanism and empirical evidence on new-type urbanization to narrow the urban–rural income gap: Evidence from China’s provincial data

**DOI:** 10.1371/journal.pone.0358432

**Published:** 2026-09-14

**Authors:** Na Zhao

The affiliation of the author is incorrect. The correct affiliation is not indicated. Na Zhao is affiliated with: School of Marxism, Xi’an International Studies University, Xi’an, PR China.
