# Supplementary material for: Mechanism and empirical evidence on new-type urbanization to narrow the urban–rural income gap: Evidence from China’s provincial data
Source: PLoS One. 2024 Aug 2;19(8):e0270964. doi: 10.1371/journal.pone.0270964 (PMC11296649; doi:10.1371/journal.pone.0270964)
Supplement: S1 Appendix — (DOCX) [file pone.0270964.s001.docx]

| **Dimension** | **Characterization** | **Index weight** | **Attribute** |
| --- | --- | --- | --- |
| Economy （E）  0.1286 | E_1_：Per capita GDP  E_2_：General budget revenue of local finance | 0.0157 | +  + |
|  |  | 0.0247 |  |
|  | E_3_：Investment in fixed assets | 0.0214 | + |
|  | E_4_：Proportion of tertiary industry output value | 0.0121 | + |
|  | E_5_：Per capita disposable income of urban residents  E_6_：Average wage of on-the-job employees of urban  E_7_：Consumption level of residents | 0.0169 | +  +  + |
|  |  | 0.0164 |  |
|  |  | 0.0214 |  |
|  | I_1_：Domestic patent applications  I_2_：R & D expenditure of Industrial Enterprises | 0.0573 | + |
|  |  | 0.0468 | + |
| Innovation（I）  0.2827 | I_3_：Technology market turnover  I_4_：Private industrial enterprises  I_5_：Legal entities in information transmission, software and information technology services  I_6_：New product development projects | 0.0824 | + |
|  |  | 0.0398 | + |
|  |  | 0.0477 | + |
|  |  | 0.0087 | + |
| Harmonization  （C）  0.1347 | C_1_：Proportion of urban population  C_2_：Urban registered unemployment rate  C_3_：Per capita income ratio of urban and rural residents | 0.0255 | + |
|  |  | 0.0043 | - |
|  |  | 0.0040 | - |
|  | C_4_：Per capita consumption expenditure ratio of urban and rural residents  C_5_：Urban residents with minimum living security | 0.0143 | - |
|  |  | 0.0085 | - |
|  | C_6_：Rural residents with minimum living security  C_7_：Urban employment | 0.0246 | - |
|  |  | 0.0536 | + |
| Green  （G）  0.2964 | G_1_：Green space coverage rate | 0.0685 | + |
|  | G_2_：Harmless treatment rate of domestic waste | 0.0501 | + |
|  | G_3_：Forest coverage  G_4_：Per capita park green space area | 0.0582 | + |
|  |  | 0.0858 | + |
|  | G_5_：Wastewater discharge | 0.0070 | - |
|  | G_6_：SO_2_ emission  G_7_：Completed investment in industrial pollution control | 0.0099 | - |
|  |  | 0.0170 | + |
| Openness  （O）  0.0652 | O_1_：Foreign-invested enterprises | 0.0142 | + |
|  | O_2_：Import and export volume of the place | 0.0142 | + |
|  | O_3_: Foreign exchange from international tourism  O_4_：Registered capital of foreign-invested enterprises  O_5_：Import and export of foreign-invested enterprises | 0.0074 | + |
|  |  | 0.0136 | + |
|  |  | 0.0159 | + |
| Sharing  （S）  0.0924 | S_1_： Public finance expenditure on General Services  S_2_： Health technicians  S_3_： Per capita education expenditure  S_4_： Public library institutions  S_5_： Public toilets  S_6_： Urban Road area per capita  S_7_： Public transport vehicles per 10000 people  S_8_： General public service of local finance  S_9_： Urban medical insurance  S_10_：Employees participating in endowment insurance | 0.0224 | + |
|  |  | 0.0278 | + |
|  |  | 0.0024 | + |
|  |  | 0.0110 | + |
|  |  | 0.0052 | + |
|  |  | 0.0051 | + |
|  |  | 0.0058 | + |
|  |  | 0.0055 | + |
|  |  | 0.0026 | + |
|  |  | 0.0046 | + |

**S1 Table. New-type urbanization development quality measurement index system.**

**S2 Table. Measurement results of new-type urbanization development in Chinese provinces.**

| **Year**  **Province** | **2008** | **2009** | **2010** | **2011** | **2012** | **2013** | **2014** | **2015** | **2016** | **2017** | **Mean value** |
| --- | --- | --- | --- | --- | --- | --- | --- | --- | --- | --- | --- |
| **Guangdong** | 80.05 | 80.26 | 95.50 | 106.53 | 117.03 | 126.97 | 131.62 | 139.22 | 147.40 | 169.91 | 119.45 |
| **Jiangsu** | 56.59 | 59.03 | 72.79 | 84.96 | 94.06 | 94.76 | 98.57 | 104.94 | 111.26 | 123.33 | 90.03 |
| **Beijing** | 42.69 | 45.46 | 52.87 | 60.84 | 69.07 | 79.51 | 83.81 | 90.00 | 96.21 | 105.86 | 72.63 |
| **Shanghai** | 47.23 | 48.06 | 55.90 | 62.03 | 64.37 | 66.42 | 72.00 | 76.99 | 82.48 | 88.37 | 66.38 |
| **Zhejiang** | 36.23 | 38.95 | 44.93 | 50.72 | 57.56 | 62.16 | 65.83 | 71.85 | 74.18 | 82.83 | 58.52 |
| **Shandong** | 32.08 | 33.20 | 38.37 | 43.90 | 48.23 | 51.36 | 57.17 | 59.20 | 65.50 | 72.19 | 50.12 |
| **Fujian** | 19.11 | 19.96 | 22.97 | 26.54 | 30.06 | 32.45 | 34.80 | 38.19 | 40.74 | 45.45 | 31.03 |
| **Liaoning** | 21.96 | 23.14 | 25.35 | 28.21 | 31.67 | 32.42 | 32.66 | 31.69 | 32.28 | 35.49 | 29.49 |
| **Tianjin** | 18.09 | 18.24 | 21.00 | 24.89 | 27.49 | 30.92 | 33.73 | 36.29 | 38.33 | 40.09 | 28.91 |
| **Hubei** | 12.42 | 14.31 | 16.22 | 18.34 | 21.56 | 25.82 | 30.41 | 35.17 | 39.37 | 43.87 | 25.75 |
| **Sichuan** | 13.06 | 14.98 | 16.93 | 19.43 | 23.06 | 24.48 | 26.86 | 28.55 | 31.01 | 35.56 | 23.39 |
| **Henan** | 12.29 | 13.29 | 14.39 | 17.18 | 20.28 | 23.13 | 25.66 | 27.76 | 31.81 | 35.49 | 22.13 |
| **Anhui** | 11.32 | 12.23 | 14.04 | 17.16 | 20.31 | 23.46 | 25.01 | 27.45 | 30.50 | 35.68 | 21.72 |
| **Shaanxi** | 9.24 | 10.82 | 12.91 | 16.07 | 19.46 | 23.76 | 26.36 | 28.75 | 31.29 | 34.51 | 21.32 |
| **Hebei** | 12.59 | 13.87 | 15.02 | 17.17 | 18.58 | 20.81 | 22.68 | 23.36 | 24.44 | 28.93 | 19.74 |
| **Hunan** | 11.14 | 12.25 | 13.69 | 15.42 | 17.61 | 19.00 | 20.71 | 22.46 | 24.41 | 29.58 | 18.63 |
| **Chongqing** | 7.96 | 8.77 | 10.61 | 13.17 | 15.86 | 18.92 | 22.23 | 22.61 | 25.40 | 26.53 | 17.21 |
| **Jiangxi** | 8.96 | 9.57 | 11.09 | 12.88 | 14.19 | 15.48 | 16.95 | 18.92 | 21.29 | 24.18 | 15.35 |
| **Heilongjiang** | 11.21 | 11.69 | 12.29 | 13.79 | 14.88 | 15.90 | 16.26 | 16.19 | 16.89 | 18.15 | 14.72 |
| **Inner Mongolia** | 8.94 | 9.82 | 10.41 | 12.45 | 14.03 | 16.02 | 17.40 | 16.80 | 17.97 | 19.51 | 14.33 |
| **Guangxi** | 8.01 | 8.96 | 9.84 | 11.42 | 12.64 | 13.81 | 15.01 | 16.63 | 18.20 | 20.28 | 13.48 |
| **Shanxi** | 9.39 | 9.49 | 10.06 | 11.18 | 12.90 | 14.89 | 13.95 | 14.84 | 16.27 | 18.85 | 13.18 |
| **Yunnan** | 7.55 | 8.13 | 9.07 | 10.42 | 12.35 | 13.84 | 14.84 | 16.25 | 18.13 | 21.24 | 13.18 |
| **Jilin** | 8.63 | 9.18 | 9.92 | 10.94 | 11.96 | 13.54 | 13.78 | 14.29 | 15.65 | 17.19 | 12.51 |
| **Xinjiang** | 6.60 | 6.69 | 7.31 | 8.77 | 9.70 | 11.13 | 12.26 | 12.84 | 13.51 | 14.86 | 10.37 |
| **Hainan** | 5.35 | 5.92 | 6.97 | 8.53 | 9.32 | 10.00 | 10.52 | 10.94 | 12.53 | 13.60 | 9.37 |
| **Guizhou** | 5.55 | 5.15 | 5.78 | 6.98 | 8.35 | 9.71 | 10.76 | 11.95 | 12.80 | 15.17 | 9.22 |
| **Gansu** | 4.74 | 5.26 | 5.82 | 6.68 | 8.34 | 9.64 | 10.48 | 10.73 | 11.97 | 12.81 | 8.65 |
| **Ningxia** | 4.63 | 4.62 | 5.84 | 6.26 | 6.78 | 7.78 | 8.87 | 9.02 | 10.15 | 10.99 | 7.50 |
| **Qinghai** | 4.87 | 5.20 | 5.60 | 6.51 | 6.94 | 7.35 | 8.35 | 8.97 | 9.65 | 10.31 | 7.37 |

**S3 Table. Measurement results of urban-rural income gap: urban-rural income ratio.**

| **Year**  **Province** | **2008** | **2009** | **2010** | **2011** | **2012** | **2013** | **2014** | **2015** | **2016** | **2017** |
| --- | --- | --- | --- | --- | --- | --- | --- | --- | --- | --- |
| **Beijing** | 2.3190 | 2.2915 | 2.1921 | 2.2329 | 2.2135 | 2.6059 | 2.5723 | 2.5699 | 2.5673 | 2.5745 |
| **Tianjin** | 2.4552 | 2.4635 | 2.4112 | 2.1849 | 2.1123 | 1.8876 | 1.8518 | 1.8451 | 1.8485 | 1.8515 |
| **Hebei** | 2.8029 | 2.8581 | 2.7297 | 2.5692 | 2.5421 | 2.4192 | 2.3700 | 2.3666 | 2.3700 | 2.3715 |
| **Shanghai** | 2.3317 | 2.3102 | 2.2777 | 2.2568 | 2.2573 | 2.3364 | 2.3047 | 2.2823 | 2.2606 | 2.2496 |
| **Jiangsu** | 2.5392 | 2.5678 | 2.5163 | 2.4378 | 2.4321 | 2.3360 | 2.2961 | 2.2867 | 2.2806 | 2.2769 |
| **Zhejiang** | 2.4548 | 2.4593 | 2.4206 | 2.3695 | 2.3743 | 2.1196 | 2.0850 | 2.0693 | 2.0658 | 2.0541 |
| **Fujian** | 2.8988 | 2.9306 | 2.9328 | 2.8373 | 2.8148 | 2.4703 | 2.4286 | 2.4125 | 2.4011 | 2.3876 |
| **Shandong** | 2.8903 | 2.9109 | 2.8534 | 2.7321 | 2.7264 | 2.5155 | 2.4593 | 2.4396 | 2.4374 | 2.4336 |
| **Guangdong** | 3.0834 | 3.1236 | 3.0288 | 2.8701 | 2.8670 | 2.6688 | 2.6253 | 2.6015 | 2.5967 | 2.5967 |
| **Hainan** | 2.8719 | 2.8983 | 2.9535 | 2.8497 | 2.8237 | 2.5463 | 2.4703 | 2.4275 | 2.4026 | 2.3886 |
| **Liaoning** | 2.5810 | 2.6454 | 2.5641 | 2.4669 | 2.4748 | 2.6273 | 2.5986 | 2.5816 | 2.5524 | 2.5456 |
| **Jilin** | 2.6009 | 2.6598 | 2.4708 | 2.3697 | 2.3503 | 2.1809 | 2.1538 | 2.1985 | 2.1884 | 2.1867 |
| **Heilongjiang** | 2.3851 | 2.4134 | 2.2311 | 2.0678 | 2.0642 | 2.2253 | 2.1629 | 2.1814 | 2.1752 | 2.1671 |
| **Shanxi** | 3.2020 | 3.2979 | 3.3038 | 3.2356 | 3.2111 | 2.8000 | 2.7322 | 2.7320 | 2.7129 | 2.7005 |
| **Anhui** | 3.0911 | 3.1272 | 2.9872 | 2.9855 | 2.9361 | 2.5751 | 2.5048 | 2.4893 | 2.4876 | 2.4800 |
| **Jiangxi** | 2.7392 | 2.7629 | 2.6744 | 2.5386 | 2.5366 | 2.4337 | 2.4029 | 2.3790 | 2.3623 | 2.3560 |
| **Henan** | 2.9705 | 2.9897 | 2.8840 | 2.7551 | 2.7167 | 2.4239 | 2.3753 | 2.3566 | 2.3282 | 2.3239 |
| **Hubei** | 2.8247 | 2.8534 | 2.7534 | 2.6637 | 2.6542 | 2.3389 | 2.2907 | 2.2840 | 2.3093 | 2.3088 |
| **Hunan** | 3.0629 | 3.0728 | 2.9466 | 2.8695 | 2.8654 | 2.6972 | 2.6411 | 2.6234 | 2.6222 | 2.6243 |
| **Inner Mongolia** | 3.0997 | 3.2098 | 3.2006 | 3.0727 | 3.0416 | 2.8941 | 2.8417 | 2.8391 | 2.8405 | 2.8345 |
| **Guangxi** | 3.8333 | 3.8819 | 3.7558 | 3.6041 | 3.5360 | 2.9115 | 2.8410 | 2.7904 | 2.7342 | 2.6932 |
| **Chongqing** | 3.4820 | 3.5166 | 3.3226 | 3.1248 | 3.1108 | 2.7151 | 2.6499 | 2.5930 | 2.5639 | 2.5474 |
| **Sichuan** | 3.0655 | 3.1015 | 3.0394 | 2.9206 | 2.9004 | 2.6522 | 2.5925 | 2.5573 | 2.5292 | 2.5131 |
| **Guizhou** | 4.2042 | 4.2798 | 4.0735 | 3.9791 | 3.9345 | 3.4869 | 3.3799 | 3.3275 | 3.3055 | 3.2788 |
| **Yunnan** | 4.2707 | 4.2810 | 4.0649 | 3.9338 | 3.8908 | 3.3405 | 3.2589 | 3.1998 | 3.1720 | 3.1429 |
| **Shaanxi** | 4.0994 | 4.1101 | 3.8234 | 3.6288 | 3.5981 | 3.1508 | 3.0717 | 3.0407 | 3.0267 | 3.0016 |
| **Gansu** | 4.0272 | 4.0032 | 3.8510 | 3.8340 | 3.8070 | 3.5560 | 3.4738 | 3.4265 | 3.4456 | 3.4377 |
| **Qinghai** | 3.8026 | 3.7929 | 3.5869 | 3.3858 | 3.2746 | 3.1497 | 3.0629 | 3.0935 | 3.0882 | 3.0826 |
| **Ningxia** | 3.5127 | 3.4643 | 3.2823 | 3.2493 | 3.2088 | 2.8262 | 2.7687 | 2.7620 | 2.7562 | 2.7447 |
| **Xinjiang** | 3.2636 | 3.1566 | 2.9388 | 2.8506 | 2.8029 | 2.6880 | 2.6610 | 2.7877 | 2.7951 | 2.7862 |

**S4 Table. Measurement results of urban-rural income gap: Theil index.**

| **Year**  **Province** | **2008** | **2009** | **2010** | **2011** | **2012** | **2013** | **2014** | **2015** | **2016** | **2017** |
| --- | --- | --- | --- | --- | --- | --- | --- | --- | --- | --- |
| **Beijing** | 0.0298 | 0.0290 | 0.0250 | 0.0255 | 0.0250 | 0.0332 | 0.0324 | 0.0321 | 0.0319 | 0.0321 |
| **Tianjin** | 0.0487 | 0.0474 | 0.0426 | 0.0339 | 0.0299 | 0.0223 | 0.0208 | 0.0203 | 0.0201 | 0.0201 |
| **Hebei** | 0.1268 | 0.1294 | 0.1185 | 0.1046 | 0.1014 | 0.0906 | 0.0857 | 0.0835 | 0.0817 | 0.0799 |
| **Shanghai** | 0.0230 | 0.0227 | 0.0208 | 0.0204 | 0.0204 | 0.0212 | 0.0207 | 0.0240 | 0.0231 | 0.0232 |
| **Jiangsu** | 0.0924 | 0.0925 | 0.0816 | 0.0749 | 0.0729 | 0.0657 | 0.0619 | 0.0595 | 0.0575 | 0.0557 |
| **Zhejiang** | 0.0822 | 0.0821 | 0.0743 | 0.0703 | 0.0692 | 0.0534 | 0.0504 | 0.0485 | 0.0470 | 0.0452 |
| **Fujian** | 0.1186 | 0.1167 | 0.1129 | 0.1052 | 0.1009 | 0.0786 | 0.0745 | 0.0724 | 0.0702 | 0.0676 |
| **Shandong** | 0.1269 | 0.1273 | 0.1211 | 0.1106 | 0.1080 | 0.0915 | 0.0860 | 0.0820 | 0.0791 | 0.0766 |
| **Guangdong** | 0.1066 | 0.1084 | 0.0969 | 0.0888 | 0.0866 | 0.0766 | 0.0741 | 0.0715 | 0.0703 | 0.0690 |
| **Hainan** | 0.1249 | 0.1251 | 0.1279 | 0.1195 | 0.1161 | 0.0950 | 0.0882 | 0.0837 | 0.0798 | 0.0774 |
| **Liaoning** | 0.0863 | 0.0896 | 0.0819 | 0.0732 | 0.0709 | 0.0772 | 0.0747 | 0.0732 | 0.0718 | 0.0712 |
| **Jilin** | 0.0982 | 0.1021 | 0.0889 | 0.0816 | 0.0799 | 0.0673 | 0.0648 | 0.0675 | 0.0662 | 0.0654 |
| **Heilongjiang** | 0.0804 | 0.0822 | 0.0694 | 0.0573 | 0.0567 | 0.0673 | 0.0624 | 0.0629 | 0.0620 | 0.0613 |
| **Shanxi** | 0.1535 | 0.1588 | 0.1552 | 0.1473 | 0.1424 | 0.1128 | 0.1063 | 0.1042 | 0.1010 | 0.0982 |
| **Anhui** | 0.1514 | 0.1524 | 0.1404 | 0.1379 | 0.1319 | 0.1029 | 0.0961 | 0.0935 | 0.0917 | 0.0893 |
| **Jiangxi** | 0.1220 | 0.1224 | 0.1145 | 0.1021 | 0.1003 | 0.0910 | 0.0874 | 0.0842 | 0.0814 | 0.0793 |
| **Henan** | 0.1450 | 0.1456 | 0.1362 | 0.1240 | 0.1193 | 0.0942 | 0.0893 | 0.0867 | 0.0832 | 0.0814 |
| **Hubei** | 0.1251 | 0.1263 | 0.1139 | 0.1045 | 0.1014 | 0.0782 | 0.0736 | 0.0718 | 0.0721 | 0.0706 |
| **Hunan** | 0.1473 | 0.1467 | 0.1368 | 0.1287 | 0.1264 | 0.1120 | 0.1062 | 0.1029 | 0.1003 | 0.0977 |
| **Inner Mongolia** | 0.1342 | 0.1375 | 0.1322 | 0.1222 | 0.1178 | 0.1073 | 0.1026 | 0.1009 | 0.0991 | 0.0970 |
| **Guangxi** | 0.2115 | 0.2131 | 0.2026 | 0.1883 | 0.1802 | 0.1323 | 0.1254 | 0.1202 | 0.1147 | 0.1102 |
| **Chongqing** | 0.1624 | 0.1604 | 0.1454 | 0.1288 | 0.1235 | 0.0974 | 0.0912 | 0.0856 | 0.0810 | 0.0775 |
| **Sichuan** | 0.1524 | 0.1537 | 0.1483 | 0.1367 | 0.1334 | 0.1130 | 0.1058 | 0.1017 | 0.0979 | 0.0950 |
| **Guizhou** | 0.2507 | 0.2567 | 0.2390 | 0.2311 | 0.2265 | 0.1896 | 0.1746 | 0.1677 | 0.1626 | 0.1574 |
| **Yunnan** | 0.2519 | 0.2512 | 0.2342 | 0.2212 | 0.2136 | 0.1710 | 0.1630 | 0.1562 | 0.1515 | 0.1466 |
| **Shaanxi** | 0.2214 | 0.2184 | 0.1944 | 0.1782 | 0.1694 | 0.1384 | 0.1307 | 0.1260 | 0.1222 | 0.1176 |
| **Gansu** | 0.2329 | 0.2293 | 0.2160 | 0.2132 | 0.2086 | 0.1878 | 0.1791 | 0.1731 | 0.1716 | 0.1676 |
| **Qinghai** | 0.2044 | 0.2012 | 0.1812 | 0.1643 | 0.1543 | 0.1439 | 0.1356 | 0.1365 | 0.1337 | 0.1305 |
| **Ningxia** | 0.1756 | 0.1701 | 0.1541 | 0.1477 | 0.1434 | 0.1155 | 0.1090 | 0.1058 | 0.1036 | 0.1000 |
| **Xinjiang** | 0.1661 | 0.1574 | 0.1366 | 0.1290 | 0.1248 | 0.1152 | 0.1114 | 0.1197 | 0.1188 | 0.1167 |

**S5 Table. Effect test.**

| Dependent Variable: GT | | |  |  |
| --- | --- | --- | --- | --- |
| Method: Panel Generalized Method of Moments | | | | |
| Transformation: First Differences | | |  |  |
| Date: 08/02/19 Time: 12:39 | | |  |  |
| Sample (adjusted): 2010 2017 | | |  |  |
| Periods included: 8 | |  |  |  |
| Cross-sections included: 8 | | |  |  |
| Total panel (balanced) observations: 64 | | | |  |
| White period instrument weighting matrix | | | |  |
| White period standard errors & covariance (d.f. corrected) | | | | |
| Instrument specification: @DYN(GT,-2) | | | |  |
| Constant added to instrument list | | | |  |
|  |  |  |  |  |
| Variable | Coefficient | Std. Error | t-Statistic | Prob. |
|  |  |  |  |  |
| GT(-1) | 2.267271 | 36.204 | 0.062625 | 0.9503 |
| LNUQ | -0.42522 | 4.721861 | -0.09005 | 0.9286 |
| LM | 0.253002 | 3.256996 | 0.077679 | 0.9384 |
| STRR | -8.62212 | 100.5768 | -0.08573 | 0.932 |
| STRH | -2.02504 | 24.18262 | -0.08374 | 0.9336 |
| PB | -16.9165 | 243.3386 | -0.06952 | 0.9448 |
| LNPGDP | -0.51567 | 10.91927 | -0.04723 | 0.9625 |
| EDU | 1.035413 | 6.916729 | 0.149697 | 0.8815 |
|  |  |  |  |  |
|  | Effects Specification | |  |  |
|  |  |  |  |  |
| Cross-section fixed (first differences) | | | |  |
|  |  |  |  |  |
| Mean dependent var | -0.00482 | S.D. dependent var | | 0.00535 |
| S.E. of regression | 0.228503 | Sum squared resid | | 2.923952 |
| J-statistic | 8.16E-10 | Instrument rank | | 8 |
